# Acute Pulmonary Embolism Decreases Adenosine Plasma Levels in Anesthetized Pigs

**DOI:** 10.5402/2011/750301

**Published:** 2011-04-26

**Authors:** François Kerbaul, Youlet By, Vlad Gariboldi, Choukri Mekkaoui, Pierre Fesler, Frédéric Collart, Serge Brimioulle, Yves Jammes, Jean Ruf, Régis Guieu

**Affiliations:** ^1^Laboratory of Hemodynamic and Cardiovascular Mechanisms, and Departments of Intensive Care, AP-HM, 13385 Marseille Cedex 05, France; ^2^UMR MD2 P2COE, Université de la Méditerranée, Faculté de Médecine, 13385 Marseille Cedex 05, France; ^3^Department of Cardiac Surgery, AP-HM, 13385 Marseille Cedex 05, France; ^4^Free University of Brussels, B-1070 Brussels, Belgium; ^5^Laboratory of Biochemistry, Timone Hospital, AP-HM, 13385 Marseille Cedex 05, France

## Abstract

Adenosine plays a role in pulmonary arterial (PA) resistance due to its vasodilator properties. However, the behavior of adenosine plasma levels (APLs) during pulmonary embolism remains unknown. We investigated the effects of gradual pulmonary embolism on right ventricular (RV) contractility and PA coupling and on APLs in an piglet experimental model of RV failure. PA distal resistance by pressure-flow relationships and pulmonary vascular impedance were measured. RV contractility was determined by the end-systolic pressure-volume relationship (Ees), PA effective elastance by the end-diastolic to end-systolic relationship (Ea), and RV-PA coupling efficiency by the Ees/Ea ratio. APLs were measured before and during gradual pulmonary embolization. PA embolism increased PA resistance and elastance, increased Ea from 1.08 ± 0.15 to 5.62 ± 0.32 mmHg/mL, decreased Ees from 1.82 ± 0.10 to 1.20 ± 0.23 mmHg/mL, and decreased Ees/Ea from 1.69 ± 0.15 to 0.21 ± 0.07. APLs decreased from 2.7 ± 0.26 to 1.3 ± 0.12 *μ*M in the systemic bed and from 4.03 ± 0.63 to 2.51 ± 0.58 *μ*M in the pulmonary bed during embolism procedure. Pulmonary embolism worsens PA hemodynamics and RV-PA coupling. APLs were reduced, both in the systemic and in the pulmonary bed, leading then to pulmonary vasoconstriction.

## 1. Introduction

It is generally assumed that acute pulmonary embolism-induced pulmonary hypertension results from the interaction of main factors such as mechanical obstruction of pulmonary vessels associated with PA constriction [1]. In this setting, RV outflow impedance is suddenly increased [2], RV ejection fraction is impaired, and the RV is enlarged [3]. Thus, both systolic and diastolic functions are impaired, which may cause or precipitate circulatory failure in critically ill patients. Experimental models of acute pulmonary embolism with autologous clots have also been reported [2–6]. Acute pulmonary embolism-induced pulmonary hypertension was shown to persistently deteriorate RV contractility by increasing RV afterload, thereby providing a rationale for inotropic support [4]. Other factors such as PA constriction attributable to neurogenic reflex and hypoxia and release of vasoconstrictors by activated platelets, leukocytes, and endothelial and lung cells could be involved [1]. Among these, adenosine (ADO), a purine nucleoside released by vascular endothelium and myocytes, may be implicated in the control of healthy and pathologic pulmonary circulation [7, 8]. ADO has strong vasodilatory properties via the activation of G-coupled membrane receptors, namely, A1, A2A, A2B or A3 depending on their affinity for ADO or agonists and primary sequence [9]. Thus extracellular ADO concentration is crucial for the control of systemic and pulmonary blood pressure. High concentration of ADO can activate, low affinity adenosine receptors, facilitating vasodilation. However, to our knowledge, there is no data on the behavior of endogenous ADO plasma levels (APLs) during acute pulmonary hypertension following pulmonary embolization. 

Therefore, our purpose was to evaluate RV performance, pulmonary hemodynamics, RV-vascular coupling, and their correlation to APLs in that model of RV failure. To better identify and quantify the PA and RV components of the experimental model and of the pharmacologic interventions, we will assess the pulmonary circulation by PA pressure-flow curves and PA impedance and the ventricular function by RV end-systolic elastance and RV-PA coupling efficiency [10].

## 2. Materials and Methods

All experiments were approved by the Animal Ethics Committee of the University School of Medicine and were done in accordance with the “Guiding Principles in the Care and Use of Animals” of the American Physiological Society.

### 2.1. Preparation

The study included 10 piétrain pigs (mean weight 40 kg) premedicated with midazolam 0.2 mg·kg^−1^ and ketamine 20 mg·kg^−1^ *i.m.*, anesthetized with midazolam *i.v.* 0.2 mg·kg^−1^ and 0.2 mg·kg^−1^·h^−1^, and paralyzed for thoracotomy with vecuronium bromide *i.v.* 1 mg·kg^−1^ and 2 mg·kg^−1^·h^−1^. Sufentanil 0.5 *μ*g·kg^−1^ *i.v*. was given at time of induction and again at beginning of surgery, and 10–20 *μ*g boluses were added to prevent increases in heart rate or blood pressure. Other details of the preparation have been published previously in [10, 11]. Briefly, the pigs were initially ventilated with an inspired oxygen fraction of 0.4, tidal volume of 12 mL/kg, a respiratory rate to achieve a PaCO_2_ of 35–40 mmHg, and a plateau pressure below 30 cm of water. A positive end-expiratory pressure of 5 cm of water was used to avoid atelectasis formation. A PA catheter (131H-7F, Baxter-Edwards, Irvine, Calif) was inserted, and normal saline was infused to maintain the occluded PA pressure between 5 and 10 mmHg. A balloon catheter (Percor, Datascope, Paramus, NJ) was advanced into the inferior vena cava to decrease cardiac output by reducing venous return. A median sternotomy was performed, and a 16 to 24 mm ultrasonic flow probe (T206, Transonic, Ithaca, NY) was positioned around the main pulmonary artery. Manometer-tipped catheters (SPC 350, Millar, Houston, Tex) were introduced into the right ventricle and proximal pulmonary artery. Then, chest was closed, but no attempt was made to restore a negative intrathoracic pressure.

### 2.2. Data Analysis

Instantaneous pressures and flow were sampled at 200 Hz. PA resistance was assessed by pressure-flow relationships obtained by rapid flow reduction. PA pressure values were interpolated at flows of 2 and 4 L/min/m^2^ from individual regressions and were averaged to obtain composite pressure-flow plots. A single beat method was applied to compute RV end-systolic elastance (Ees) as the slope of the end-systolic pressure-volume line, PA effective elastance (Ea) as the absolute slope of the end-systolic to end-diastolic line, and ventriculoarterial coupling efficiency as the Ees/Ea ratio. This method has been validated during variations of preload, afterload, and inotropism and has proven reliable in conditions of pulmonary hypertension and RV failure [11–13].

Pulmonary vascular impedance spectrum (PVZ) was calculated from the Fourier series expressions for pressure and flow signals as previously reported in [10]. Five end-expiratory heartbeats are analyzed for each data-collection interval. The PVZ modulus was computed as the ratio between pressure and flow moduli, and its phase is computed as the difference between flow and pressure phases. The impedance at 0 Hz (Zo) was taken as the total resistance (Ppa/Q), and characteristic impedance (Zc) was calculated as the average of impedance moduli between 2 and 15 Hz. From the PVZ spectra were also derived the first harmonic modulus (Z1) and the first harmonic phase angle (Ph_1_).

### 2.3. Protocol

Each data set included flow and pressure values collected at steady state for calculation of impedance and RV-PA coupling and collected during a flow reduction maneuver for construction of pressure-flow relations. A first data set was obtained after 30 minutes of postoperative stabilization, during a transient period of apnea following the end of expiratory phase. The same procedure was repeated 30 min after each injection of autologous blood clots. For this purpose, a 250 mL blood sample, collected before sternotomy, was allowed to clot in a beaker and was cut into 3 to 5 mm pieces for injection. A large bore polyethylene cannula (ID 12 French) was inserted into the left external jugular vein, and blood clot pieces were injected by an irrigation syringe over 30 min. Embolization was carried out progressively until reached 30–35 mmHg in a first step, Ppa 40–45 mmHg in a second step, and 50–55 mmHg in a third step. The Ppa stabilized after 30 min at a level that was in general 3–5 mmHg below the value achieved at the end of embolization. A complete set of measurements was also taken at each step. Thereafter, for each level of Ppa, arterial blood gases and APLs levels were also recorded.

At the end of the study, each animal was killed by intravenous injection of potassium chloride, under deep intravenous anesthesia.

### 2.4. Samples Collection

ADO was measured at baseline and for each step of pulmonary embolism (just 1 minute before hemodynamic measurements). Blood samples were taken from both carotid and pulmonary arteries. ADO sample collection and treatment have been previously described in [7, 8]. Briefly, the lumens of the arterial and pulmonary catheters were washed out and filled with a solution of 1 mL of papaverine and 1 mL of dipyridamole, injected through the lateral entry of a 3-way stopcock just prior to blood sampling.

### 2.5. ADO Assay

Samples were immediately centrifuged, deproteinated (perchloric acid, 70%), and lyophilized, before being analyzed by chromatography as previously described in [14, 15].

### 2.6. Statistical Analysis

Hemodynamic, APLs, and metabolic variables were analyzed by a repeated-measures analysis of variance followed by Fisher's protected *t*-tests. *P* < .05 was considered statistically significant. Spearman correlation was performed between mean PA pressure, mean PA elastance, and APLs. Data were expressed as mean (s.e.m). Statistical analysis was performed using SPSS software, version 11.5 for Windows.

## 3. Results

### 3.1. Baseline

**Figure 1:**
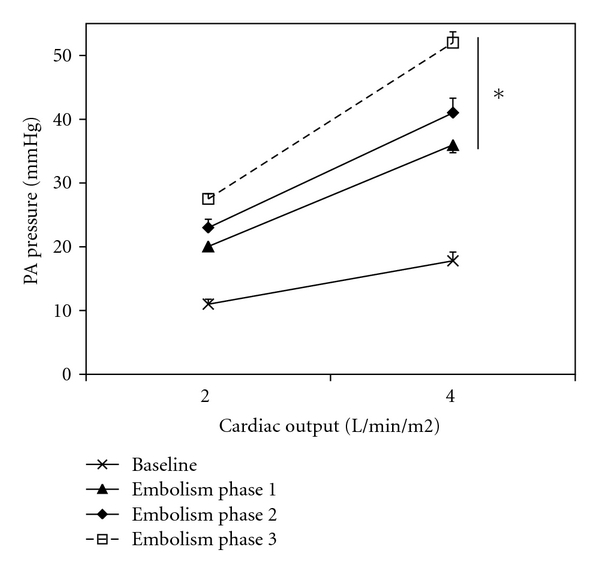
Pulmonary arterial (PA) pressure versus cardiac output plots at baseline and after pulmonary embolism (highest level) (mean ± s.e.m, *n* = 10). Pressure-flow plots shifted upwards with embolism. *P < .05 versus baseline.

Hemodynamic and blood gas data are reported in Tables 1 and 2. APLs were significantly higher in the distal pulmonary artery than in the carotid artery. Ppa/Q plots were linear with correlation coefficients from 0.96 to 1. Representative Ppa/Q plots are shown in Figure 1.

### 3.2. Effects of Pulmonary Embolism

**Figure 2:**
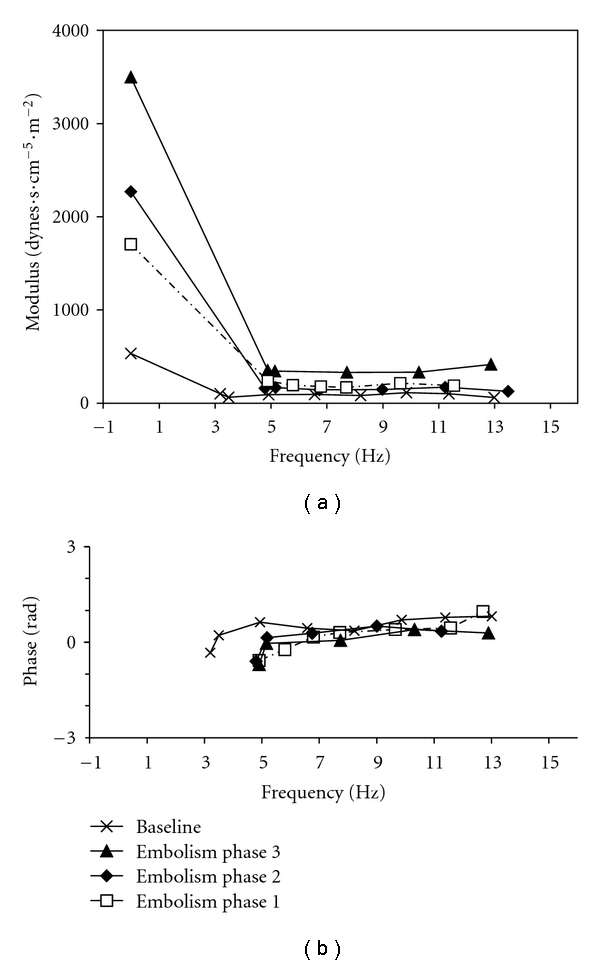
Pulmonary vascular impedance spectra at baseline and after gradual pulmonary embolism (mean ± s.e.m, *n* = 10). The impedance spectrum shifted upwards during embolism.

**Figure 3:**
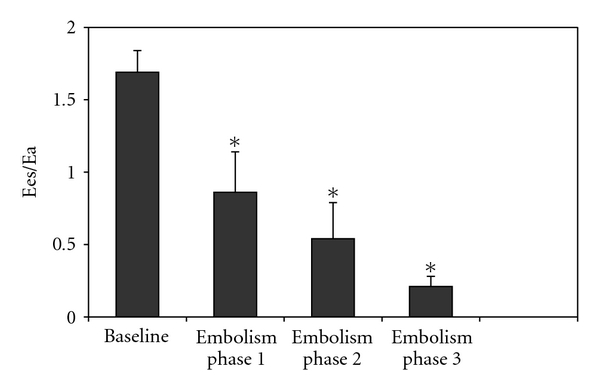
Right ventriculopulmonary arterial (PA) coupling efficiency assessed as the ratio of ventricular end-systolic elastance (Ees) to effective arterial elastance (Ea) at baseline and after gradual pulmonary embolism (mean ± s.e.m, *n* = 10). Gradual embolism markedly and persistently decreased Ees/Ea. *P < .05 versus baseline.

PA embolism increased heart rate, PA, and right atrial pressures and decreased progressively cardiac output. Systemic arterial pressure was significantly reduced only at the third step (Table 1). Pressure-flow plots were shifted upwards (Figure 1). Impedance spectra were also shifted upwards, with increased Zo and Zc, and more negative low-frequency phases (Figure 2). Ea increased from about 1.08 to 5.62 mmHg/mL, Ees initially increased in the first two steps but decreased from about 1.82 to 1.20 mmHg/mL, and Ees/Ea markedly decreased from about 1.69 to 0.21 at the third step of embolism (Figure 3). Lung embolization was associated with marked decreases in arterial pH, O_2_ tension, and APLs (Table 3) and with increases in arterial CO_2_ tension (Table 2). In every instance, APLs were higher in the distal pulmonary arteries than in the systemic circulation. Correlation between mean PA pressures, mean Ea and pulmonary artery APLs at all levels was negative (*r* = −0.64; *P* < .01 and *r* = −0.79, *P* < .01, resp.).

## 4. Discussion

The present results (I) demonstrate that an acute pressure overload following pulmonary embolism by autologous clots induces a RV failure, (II) show that the RV-PA uncoupling is due both to an increase in PA resistance and elastance and to a depression of RV contractility, and (III) suggest that adenosine release by pulmonary vascular endothelium is reduced following pulmonary embolism.

### 4.1. Pulmonary Arterial Changes

In addition to PA pressure and resistance, we used flow-pressure curves to better detect resistance changes due to distal small muscular arteries [16] and pulmonary vascular impedance to assess elastic changes due to large proximal arteries [17]. Zc is the ratio of inertance versus compliance of the proximal pulmonary arteries and varies directly with the elastic modulus of the vessel and indirectly with its cross-sectional area [17]. In our study, Zc remained abnormal and indicated an increased elastance (increased stiffness decreased compliance) of proximal vessels, contributing to increased wave reflections and to an increased RV afterload. Similar Zc changes have also been reported previously after PA constriction [11]. This persistence of changes could result from a sympathetic stimulation due to the transient low cardiac output and hypotension [10] or from an activation of stretch receptors due to the PA vasoconstriction [18]. 

It was reported that transient PA hypertension following autologous blood clot embolic pulmonary hypertension was associated with an increase of pulmonary vascular tone and PVZ spectra in mammals [2]. Pulmonary embolism induced by autologous blood clots in pigs has been reported to increase PVR, Zo, Zc, and phase angle negativity [4]. Our results concur. But pulmonary embolism induced by 150–200 *μ*m glass beads or in dogs was associated with a decrease of Zc [19]. A decrease in Zc could be explained by dilatation of the PA tree. These discrepancies could be due to the different types of acute pulmonary embolism (autologous clots versus glass beads).

### 4.2. Right Ventricular Changes

RV contractility was clearly increased after the first and second steps of embolization, as evidenced by a significant Ees augmentation. This feature is in agreement with Ghuysen et al.'s study [6] suggesting that increment in Ea following pulmonary embolism could be initially coupled with preload recruitment to maintain RV performance. But RV contractility remained altered after the third step of PA embolism (Ees changed from 1.82 to 1.20 mmHg/mL), while afterload remained increased suggesting that the combination of both factors results in a marked deterioration of RV-PA coupling efficiency. In contrast with Ghuysen's study, our data showed in response to several gradual afterload increases (3 steps of embolisms) that RV-PA coupling was not maintained at maximal efficiency level because of a sharp increase of Ea and mean Ppa (47 mmHg in our study versus 30 mmHg in their study). Another experimental study showed that optimal RV-PA coupling could be maintained in dogs after distal PA embolism by 0.5-mm glass beads [20]. These results are consistent with the concept that most of the load imposed on the right ventricle is contained in the proximal PA tree, so that an increase in distal resistance (Zo) will depress RV performance and output less than an increase in proximal elastance (Zc and Ea) [20]. The second discrepancy with Wauthy et al.'s experimental study could be due to the choice of animal model. Dogs are also known to have good collateral ventilation, experience less local hypoxia than pigs and then require less hypoxic pulmonary vasoconstriction and show less extensive muscularity than pigs [21]. The different types of protocols could also explain why RV coupling was preserved in a previous study when dynamic RV afterload was increased about twofold [6, 20] and why it deteriorated here when resistance was sixfold increased. The RV changes could perhaps result from RV myocardial stunning due to hypotension and RV ischemia [22] or to other yet undefined humoral, chemical, or mechanical factors. Finally, changes in coupling values obtained in our study also were comparable to those reported in patients with left ventricular failure [23].

### 4.3. APLs and Pulmonary Embolism

In every instance, APLs were higher in the distal pulmonary arteries than in the systemic circulation. The short half-life of ADO argues against the hypothesis that ADO withdrawn from the distal pulmonary artery was released in the whole circulation [24]. It supports the assumption that ADO is released essentially locally or upstream, near the sampling site, by the pulmonary vascular endothelium [8]. 

Gradual pulmonary embolization was associated with significant reductions of O_2_ tension and APLs. But there is no evidence that PaO_2_ has any effect on ADO uptake, because ADO could cross into red cells via an equilibrative transporter [24]. Therefore, pulmonary tissue ischemia due to either low flow or hypoxia should result in ADO release. This has been experimentally shown on normal canine lung tissue [25]. Although exogenous ADO was shown to inhibit hypoxic pulmonary vasoconstriction in isolated rat lungs [26], it is not clear how endogenous ADO acts in the regulation of pulmonary blood flow and hypoxic pulmonary vasoconstriction. Moreover, the correlation between mean PA pressures, mean Ea and APLs suggests that ADO could play a role in the regulation of pulmonary vascular tone. 

Elevated PA pressures may promote and accelerate pulmonary vascular wall damage [27]. Since ADO is released by endothelial cells [28], one can hypothesize that pulmonary vascular endothelium injured by embolism becomes unable to release enough ADO to stimulate low-affinity spare receptors [29] which could promote pulmonary vasodilation. Finally, it is well established that systemic vascular obstruction is followed by a strong increase in APLs, due to the ischemic process [30]. Here we found that pulmonary bed obstruction was followed by a decrease in APLs. Because it is well established that ADO reduces pulmonary resistances [8], the low concentration in APLs could contribute to an increase of PA hypertension in our model.

## 5. Study Limitations

We do not explore whether the pulmonary adenosine administration could prevent part of the abnormalities; however, this should be done in further studies.

## 6. Conclusion

Acute pulmonary embolism-induced pulmonary hypertension deteriorates right ventricular-pulmonary arterial coupling both by increasing PA resistance and elastance and by decreasing RV contractility, thereby confirming a rationale for both inotropic and pulmonary vasodilatory support. In this context, low ADO concentrations may be the result of the injured pulmonary vascular endothelium, leading then to an increase of pulmonary vasoconstriction. Finally, this study suggests that ADO infusion could be an adjuvant treatment for pulmonary embolism.

## Figures and Tables

**Table 1:** Hemodynamics before and after gradual pulmonary artery embolism.

	Baseline	Embolism 1	Embolism 2	Embolism 3
Heart rate, bpm	90 ± 8	122 ± 8^a^	135 ± 9^a^	148 ± 8^ab^
CO, L/min/m^2^	4.1 ± 0.1	3.3 ± 0.4^a^	2.7 ± 0.3^a^	1.7 ± 0.3^abc^
Psa, mmHg	110 ± 10	119 ± 11	106 ± 8	88 ± 8^abc^
Ppa, mmHg	18 ± 2	31 ± 3^a^	39 ± 3^ab^	47 ± 4^abc^
Pra, mmHg	7 ± 1	7 ± 1	9 ± 2^ab^	10 ± 1^ab^
Ppao, mmHg	7 ± 1	7 ± 1	11 ± 1^ab^	12 ± 1^ab^
Zo, dyn·s·cm^−5^·m^2^	530 ± 45	1704 ± 244^a^	2268 ± 190^ab^	3498 ± 208^abc^
Zc, dyn·s·cm^−5^·m^2^	92 ± 13	265 ± 30^a^	297 ± 59^a^	416 ± 43^abc^
F min, Hz	3.40 ± 0.25	4.90 ± 0.50^a^	4.88 ± 0.71	4.92 ± 0.53^a^
Ph1, rad	−0.32 ± 0.03	−0.57 ± 0.08^a^	−0.55 ± 0.09^a^	−0.65 ± 0.10^a^
Ees, mmHg/mL	1.82 ± 0.10	2.70 ± 0.06^a^	2.28 ± 0.42^a^	1.20 ± 0.23^abc^
Ea, mmHg/mL	1.08 ± 0.15	3.15 ± 0.13^a^	4.22 ± 0.40^ab^	5.62 ± 0.32^abc^
Ees/Ea	1.69 ± 0.15	0.86 ± 0.28^a^	0.54 ± 0.25^a^	0.21 ± 0.07^abc^
SV, mL	41 ± 3	27 ± 2^a^	18 ± 2^ab^	12 ± 3^abc^

Values are means ± SE (*n* = 10). CO: cardiac output; Psa: systemic arterial pressure; Ppa: pulmonary arterial pressure; Pra: right atrial pressure; Ppao: occluded Ppa; Zo: 0-Hz impedance; Zc: characteristic impedance; Ees: end-systolic elastance; Ea: arterial elastance. ^a^ *P* < .05 versus baseline; ^b^ *P* < .05 versus embolism 1; ^c^ *P* < .05 versus embolism 2.

**Table 2:** Respiratory parameters before and during pulmonary artery embolism.

	Baseline	Embolism 1	Embolism 2	Embolism 3
pH	7.40 ± 0.01	7.34 ± 0.02^a^	7.30 ± 0.01^ab^	7.25 ± 0.02^abc^
PaO2, mmHg	164 ± 20	120 ± 20^a^	88 ± 12^a^	51 ± 5^abc^
PaCO2, mmHg	36 ± 1	39 ± 5^a^	48 ± 5^ab^	62 ± 6^abc^

Values are means ± s.e.m (*n* = 10).

^a^ *P* < .05 versus baseline; ^b^ *P* < .05 versus embolism 1; ^c^ *P* < .05 versus embolism 2.

**Table 3:** Plasmatic values of adenosine before and during pulmonary artery embolism.

	Baseline	Embolism 1	Embolism 2	Embolism 3
Carotid artery, *μ*moL/L	2.73 ± 0.26	1.79 ± 0.26^a^	1.99 ± 0.28^a^	1.30 ± 0.12^abc^
Pulmonary artery, *μ*moL/L	4.03 ± 0.63	2.44 ± 0.42^a^	2.98 ± 0.5^a^	2.51 ± 0.58^a^

Values are means ± s.e.m (*n* = 10).

^a^ *P* < .05 versus baseline; ^b^ *P* < .05 versus embolism 1; ^c^ *P* < .05 versus embolism 2.

## Abbreviations

ADO: Adenosine
APLs: Adenosine plasma levels
Ea: Affective pulmonary arterial elastance
Ees: End-systolic right ventricular elastance
HR: Heart rate
*i.m.*: Intramuscular
*i.v.*: Intravenous
PA: Pulmonary arterial
Ppa: Mean pulmonary arterial pressure
Psa: Mean systemic arterial pressure
Q: Pulmonary artery flow
RV: Right ventricular
RV-PA: Right ventricular-vascular coupling (right ventricular-pulmonary arterial coupling).
